# High-charge electron beams from a laser-wakefield accelerator driven by a CO_2_ laser

**DOI:** 10.1038/s41598-022-10160-9

**Published:** 2022-05-18

**Authors:** Enrico Brunetti, R. Neil Campbell, Jack Lovell, Dino A. Jaroszynski

**Affiliations:** 1 SUPA, Department of Physics, University of Strathclyde, Glasgow, UK; 2 IRP Technology LLC, Corrales, NM USA

**Keywords:** Plasma-based accelerators, Laser-produced plasmas

## Abstract

Laser-wakefield accelerators (LWFAs) driven by widely available 100s TW-class near-infrared laser systems have been shown to produce GeV-level electron beams with 10s–100s pC charge in centimetre-scale plasma. As the strength of the ponderomotive force is proportional to the square of the laser wavelength, more efficient LWFAs could be realised using longer wavelength lasers. Here we present a numerical study showing that $10.6\,\upmu \hbox {m}$, sub-picosecond CO_2_ lasers with peak powers of 100–800 TW can produce high-charge electron beams, exceeding that possible from LWFAs driven by femtosecond near-infrared lasers by up to three orders of magnitude. Depending on the laser and plasma parameters, electron beams with 10s MeV to GeV energy and 1–100 nC charge can be generated in 10–200 mm long plasma or gas media without requiring external guiding. The laser-to-electron energy conversion efficiency can be up to 70% and currents of 100s kA are achievable. A CO_2_ laser driven LWFA could be useful for applications requiring compact and industrially robust accelerators and radiations sources.

## Introduction

Laser-wakefield accelerators (LWFAs) rely on the strong electrostatic fields created by an intense laser pulse propagating in underdense plasma to generate high-energy electron beams in millimetre-scale distances^1–7^. Research in this field is mostly carried out using Ti:sapphire lasers with a typical wavelength of ${0.8}\,\upmu \hbox {m}$, energy of 1–50 J and duration of 10–100 fs, and operate at a repetition rate close to 1 Hz. After interaction with a millimetre to centimetre scale plasma with densities in the range $10^{17}$–$10^{19}\,{\hbox {cm}^{-3}}$, electron beams can be generated with typical energies of 10–1000 MeV, divergence of 1–5 mrad, bunch charge of 1–100 pC, normalised emittance of 1 $\pi$ mm mrad and femtosecond bunch duration^8–10^. The highest electron beam energy reported to date is 7.8 GeV, with charge of 5 pC, obtained using a 20 cm long plasma with density of ${2 \times 10^{17}}\,{\hbox {cm}^{-3}}$ and an 850 TW laser system^11^.

The response of plasma to an intense laser pulse is a result of the ponderomotive force^12^, which has a strength proportional to the square of the laser wavelength. It has been suggested^13^ that more efficient LWFAs capable of producing electron beams with higher charge and narrower energy spread could be realised using mid-infrared lasers, but research in this area is scarce. Early experiments on plasma beat-wave acceleration used CO_2_ lasers^14,15^, and theoretical^16–18^ and experimental^19^ studies have been performed on a self-modulated LWFA driven by the CO_2_ laser system under development at the Brookhaven Accelerator Test Facility^20^, which delivers 4 J, 2 ps pulses and is expected to be upgraded to 100 fs duration and 10 J energy. Two-dimensional simulations of a LWFA driven by a few-cycle laser pulse with wavelength up to ${10}\,{\upmu }\hbox {m}$ have also been reported^21^. However, no systematic study has been carried out so far to evaluate the performance of an accelerator driven by a CO_2_ laser. Here we present results of particle-in-cell (PIC) simulations obtained using the code FBPIC^22^ to model the interaction of an intense sub-picosecond laser pulse with wavelength $\lambda _0={10.6}\,\upmu \hbox {m}$ and pre-ionised uniform plasma of varying length. We explore a wide range of laser pulse durations, waist sizes and energies for selected plasma densities with the aim of determining the highest electron beam energy and charge achievable using CO_2_ lasers that could become available in the near future^23^. Most results presented here assume no external guiding, but comparisons are made with simulations that model a LWFA in a plasma waveguide.

## Laser-wakefield acceleration at CO_2_ laser wavelengths

The operating parameters of a LWFA driven by a CO_2_ laser are different from those currently used for near-infrared lasers. At the wavelength $\lambda _0={10.6}\,\upmu \hbox {m}$, the critical plasma density is ${9.9 \times 10^{18}}\,{\hbox {cm}^{-3}}$, in contrast with ${1.7 \times 10^{21}}\,{\hbox {cm}^{-3}}$ at $\lambda _0={0.8}\,\upmu \hbox {m}$, which sets an upper limit to the plasma density $n_e$ and thus the accelerating gradient, because plasma can only produce an electric field with an amplitude up to about^24^ $E_{\max }[{\hbox {V/m}}]\approx 96 \sqrt{n_e[{\hbox {cm}^{-3}}]}$. The laser characteristics are also different. At the wavelength $\lambda _0={10.6}\,\upmu \hbox {m}$, a single cycle is about 35 fs long, therefore the attainable pulse durations are longer than routinely delivered by Ti:sapphire lasers. A LWFA operating in the strongly non-linear regime requires a normalised vector potential $a_0>1$, where $a_0 \approx 8.5\times 10^{-10}\lambda _0[\,\upmu \hbox {m}]\sqrt{I_0[{\hbox {W/cm}^2}]}$, with $I_0$ the laser intensity. As the wavelength increases, lower intensities are required to achieve the same $a_0$ value, e.g. $a_0$ for ${10.6}\,\upmu \hbox {m}$ will be 13.25 times larger than at ${0.8}\,\upmu \hbox {m}$ for the same intensity. Simulations presented here use plasma densities between ${1 \times 10^{16}}\,{\hbox {cm}^{-3}}$ and ${2 \times 10^{17}}\,{\hbox {cm}^{-3}}$, laser waist sizes between ${25}\,\upmu \hbox {m}$ and ${300}\,\upmu \hbox {m}$, pulse durations between 125 fs and 1 ps (full-width-at-half-maximum (FWHM) of the intensity), and laser energies between 0.4 J and 550 J, corresponding to $a_0$ values between 1 and 80, which is much larger than possible with a ${0.8}\,\upmu \hbox {m}$ PW laser.

The performance of a LWFA depends on the distance over which the laser intensity remains high. In vacuum, this is given by the Rayleigh (diffraction) length $z_R = \pi w_0^2 / \lambda _0$, with $w_0$ the beam waist at the focus. For $\lambda _0={10.6}\,\upmu \hbox {m}$, $z_R={0.2}\,{\hbox {mm}}$ when $w_0={25}\,\upmu \hbox {m}$ and $z_R={26.7}\,\hbox {mm}$ when $w_0={300}\,\upmu \hbox {m}$. In a plasma, an intense laser pulse can remain focused over several Rayleigh lengths when its power *P* exceeds the threshold for relativistic self-focusing^25^, which is indicated by the critical power $P_c [\hbox {GW}] = 17.4\,\omega _0^2 / \omega _p^2$, where $\omega _0$ is the laser angular frequency and $\omega _p$ is the plasma frequency. When $P \approx P_c$, plasma electrons exhibit a relativistic quiver motion that modifies the refracting index, resulting in a radial profile analogous to a focusing lens. When $P > P_c$, electrons are also expelled from the axis, forming a channel that can guide the laser. These changes in refractive index occur on the plasma frequency time-scale, therefore self-guiding is expected to be more effective when $cT_{{\mathrm {FWHM}}} \approx \lambda _p$, with $T_{{\mathrm {FWHM}}}$ the laser pulse duration and $\lambda _p$ the plasma wavelength^26^. For shorter pulse durations, guiding may not occur or may only affect the back of the pulse. For longer durations, instabilities such as self-modulation can develop. The threshold for relativistic self-focusing can be reached for a similar laser power at near and mid-infrared wavelengths, since the change in laser frequency is compensated by the different operating plasma density. However, the Rayleigh length is one order of magnitude shorter for a CO_2_ laser, suggesting that diffraction may be harder to balance for lower laser energies and small waist sizes.

Assuming stable guiding, the energy gain in a LWFA is limited by the pump depletion length^27^ or the dephasing length^28^. Pump depletion occurs when most of the laser energy has been transferred to the plasma and the laser pulse diffracts, preventing further acceleration. Dephasing occurs when electrons reach the centre of the accelerating cavity, where the fields reverse and start decelerating them. The laser pulse and accelerating cavity travel at a speed close to the laser group velocity in a plasma $v_g = c\sqrt{1 - \omega _p^2 / \omega _0^2}$. For $\lambda _0={10.6}\,\upmu \hbox {m}$ and plasma densities of $10^{16}$–$10^{17}\,{\hbox {cm}^{-3}}, v_g$ is between 0.995*c* and 0.9995*c*, which corresponds approximately to the speed of 5–15 MeV electrons. Electrons at the back of the cavity with a velocity $v_e>v_g$ can be self-injected into the cavity, where they experience the accelerating fields, and accelerate to 100s MeV energy. Depending on the plasma density they can outrun the laser pulse in relatively short distances, which effectively defines the maximum useful length of a LWFA.

## Simulation results

Particle-in-cell (PIC) simulations have been performed using the quasi-3D code FBPIC^22^ to model a linearly polarised laser beam with wavelength $\lambda _0={10.6}\,\upmu \hbox {m}$ interacting with pre-ionised plasma of varying length. The laser pulse has a $\cos ^2$ temporal shape and is focused at the plasma entrance to a beam waist $w_0$, with transverse Gaussian profile. Most results presented here use longitudinally and transversally uniform plasma, but propagation in a plasma with a radial parabolic profile has also been studied, which models a preformed plasma waveguide^29^. When modelling systems that do not strongly deviate from cylindrical symmetry, quasi-3D simulations closely reproduce full 3D PIC simulations, correctly capturing the physics governing laser-plasma interaction, such as relativistic self-focusing, while requiring less computing resources. This enabled us to explore a wide range of parameters. FBPIC also has the advantage of accurately modelling dispersion, avoiding artefacts such as incorrect dephasing length and numerical Cherenkov radiation, which can cause unphysical emittance growth^22^. On the other hand, quasi-3D codes cannot model asymmetric laser beams, in addition to plasma and electron beam features that strongly deviate from cylindrical symmetry. Further details on the simulation setup are provided in Methods.

**Figure 1:**
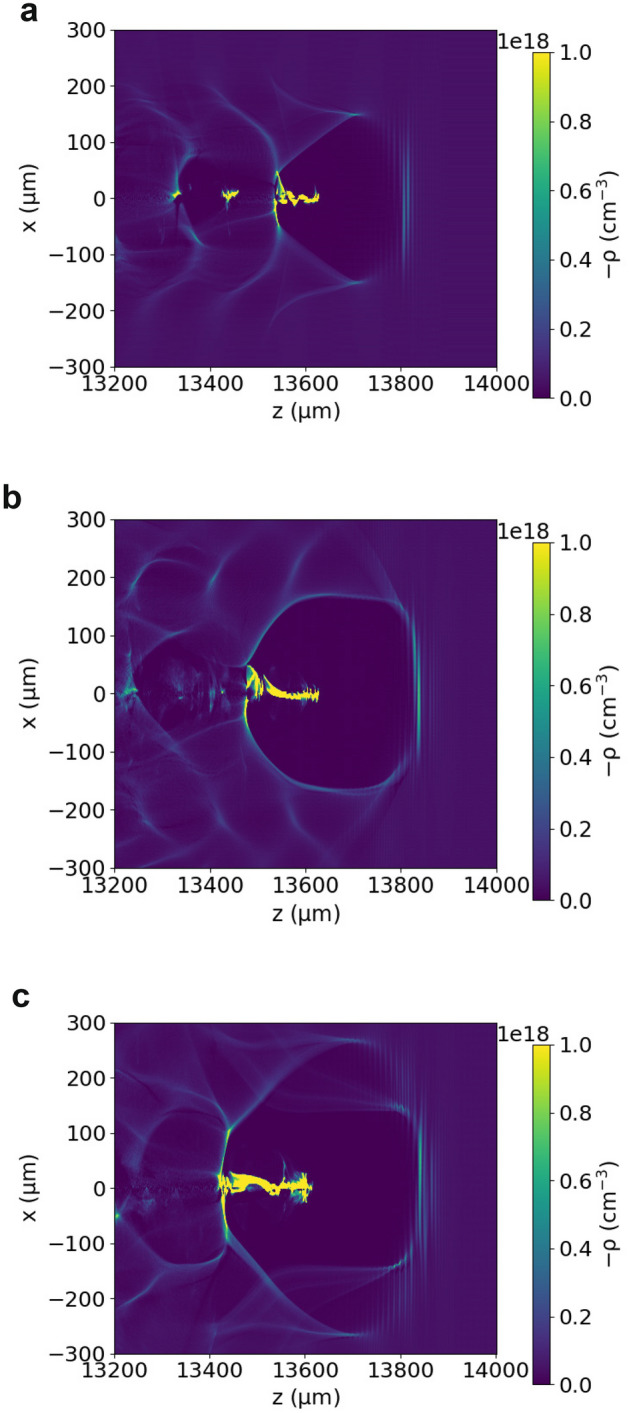
Electron density distribution created in a plasma with density of ${5 \times 10^{16}}\,{\hbox {cm}^{-3}}$ by a laser pulse with a wavelength of ${10.6}\,\upmu \hbox {m}$, normalised vector potential $a_0=5$, pulse duration of 500 fs (FWHM) and initial waist size of (**a**) ${100}\,\upmu \hbox {m}$ (**b**) ${200}\,\upmu \hbox {m}$ and (**c**) ${300}\,\upmu \hbox {m}$.

Figure 1 shows the accelerating structure after 14 mm, created behind a 500 fs duration (FWHM) laser pulse with normalised vector potential $a_0=5$ for various beam waists. The laser pulse propagates in a pre-ionised uniform plasma with density of ${5 \times 10^{16}}\,{\hbox {cm}^{-3}}$. The ponderomotive force of the laser pulse pushes electrons away from the regions of high intensity, which produces a cavity (“bubble” or “blowout”) filled with relatively immobile ions and surrounded by a “sheath” of electrons. Background plasma electrons that are trapped inside the bubble are accelerated to relativistic energies by the strong electrostatic fields arising from charge separation. The bubble shape and size depends on the plasma density, on the laser power and on the strength of the fields of the electrons trapped inside (beam loading). These parameters usually vary during propagation, which leads to bubble evolution, as will be discussed below. This can influence the injection rate and change the accelerating gradient. The combined effects produce trains of bunches or single bunches with a complex energy spectrum and usually significant sub-structure. Depending on the laser and plasma parameters, electrons injected late can form low-energy peaks or tails, or can be accelerated to high energies and account for most of the charge. Electrons injected early can form a low-charge high-energy wing, or can be decelerated back to low energies. Electrons can also be trapped inside cavities created further behind the laser pulse, but these are not considered here.

**Figure 2:**
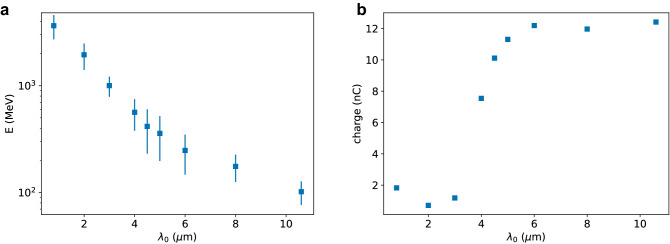
(**a**) Mean energy and (**b**) charge of electron beams produced by a laser pulse with duration of 250 fs (FWHM), waist size of ${50}\,\upmu \hbox {m}$, normalised vector potential $a_0=5$ and varying wavelength after interaction with a pre-ionised plasma with density of ${1\times10^{17}}\,{\hbox {cm}^{-3}}$. The error bars represent the r.m.s. energy spread.

The accelerator performance depends on the laser wavelength as illustrated in Fig. 2, which shows the mean energy and charge of electron beams produced by a laser pulse with a wavelength $\lambda _0$ between ${0.8}\,\upmu \hbox {m}$ and ${10.6}\,\upmu \hbox {m}$, duration $T_{FWHM}={250}\,{\hbox {fs}}$, waist size $w_0={50}\,\upmu \hbox {m}$ and $a_0=5$, which corresponds to an energy of about 550 J (2 PW) at ${0.8}\,\upmu \hbox {m}$ and 3.2 J (12 TW) at ${10.6}\,\upmu \hbox {m}$. The plasma has a uniform profile with density of ${1 \times 10^{17}}\,{\hbox {cm}^{-3}}$. For $\lambda _0={0.8}\,\upmu \hbox {m}$, the electron beam energy is about 3.6 GeV with 25% r.m.s. energy spread and a charge of 1.8 nC, after 200 mm propagation, which is slightly before dephasing. The laser-to-electron energy conversion efficiency is 1.2%. The normalised emittance is about ${15}\,{\pi\,\hbox {mm}\,\hbox{mrad}}$, the r.m.s. divergence 1 mrad and the r.m.s. bunch length ${6}\,\upmu \hbox {m}$. Electrons are injected into the bubble after about 10 mm and the corresponding acceleration gradient is about 20 GeV/m. When $\lambda _0$ is increased for fixed $a_0$, the laser energy decreases, resulting in a lower electron beam energy. The charge is also expected to decrease with laser energy, but this effect is counteracted by the stronger plasma response at longer wavelengths, resulting in a charge of about 12 nC for $\lambda _0 \gtrapprox {6}\,\upmu \hbox {m}$. The bunch length also increases, whereas the transverse beam size remains close to ${5}\,\upmu \hbox {m}$. For $\lambda _0={10.6}\,\upmu \hbox {m}$, the electron beam mean energy is 100 MeV with 21% r.m.s. energy spread and a charge of 12 nC after 9 mm propagation. The laser energy to beam energy conversion efficiency is 40%, the normalised emittance ${20}\,\pi\,\hbox {mm}\,\hbox{mrad}$, the r.m.s. divergence 20 mrad and the r.m.s. bunch length ${50}\,\upmu \hbox {m}$. Injection into the bubble occurs after about 6 mm and the corresponding acceleration gradient is about 30 GeV/m. If the laser energy is kept constant at about 550 J ($a_0=66$), a CO_2_ laser produces an electron beam with mean energy of 750 MeV, 65% r.m.s. energy spread and 530 nC charge in a 15 mm long plasma, corresponding to an efficiency of 70%. The normalised emittance is about ${750}\,\pi\,\hbox {mm}\,\hbox{mrad}$, the r.m.s. divergence 75 mrad and the r.m.s. bunch length ${170}\,\upmu \hbox {m}$. If only the high-energy peak is selected (${1.25}\,{\hbox {GeV}}<\hbox {E}<{1.45}\,{\hbox {GeV}}$), the mean energy is 1.3 GeV with 3% r.m.s. energy spread, 130 nC charge and 10 mrad r.m.s. divergence.

These results suggest that a LWFA driven by a ${10.6}\,\upmu \hbox {m}$ laser can produce electron beams with significantly higher charge than currently possible using near-infrared lasers of the same power, although the electron energy is somewhat lower and the divergence, bunch length and emittance are larger. Near-infrared lasers have the advantage of supporting higher plasma densities, which can help reduce the gap in charge. For example, a ${0.8}\,\upmu \hbox {m}$, 2 PW laser delivering pulses with 35 fs duration and 75 J energy into a plasma with a density of ${1.5 \times 10^{19}}\,{\hbox {cm}^{-3}}$ produces an electron beam with 860 MeV energy, 69% r.m.s. energy spread and 45 nC charge with an efficiency of 50%. For a high-energy selection (${1}\,\hbox {GeV}<\hbox {E}<{1.85}\,\hbox {GeV}$), the mean energy is 1.4 GeV with 17% r.m.s. energy spread and 22 nC charge. Nevertheless, this is still 5 times lower than the charge predicted for a CO_2_ laser with the same power.

In the following sections, the performance of a LWFA accelerator operating at a wavelength of ${10.6}\,\upmu \hbox {m}$ is investigated by varying the laser energy, waist size and pulse duration for selected plasma densities. Scaling laws based on the simulation results are also provided.

### Dependence on laser energy

Here we present results of PIC simulations of a LWFA driven by a CO_2_ laser with a fixed pulse duration and varying energy, for selected waist sizes. The phase-space distribution of electrons accelerated inside the first bubble to an energy $E>{30}\,\hbox {MeV}$ has been extracted when the mean energy is maximum, and properties such as the beam size, charge and energy spread have been calculated. These values describe the entire bunch, which can have a complex energy spectrum that is characterised by multiple peaks and long wings, but properties of selected quasi-monoenergetic peaks are also provided in the text.

**Figure 3:**
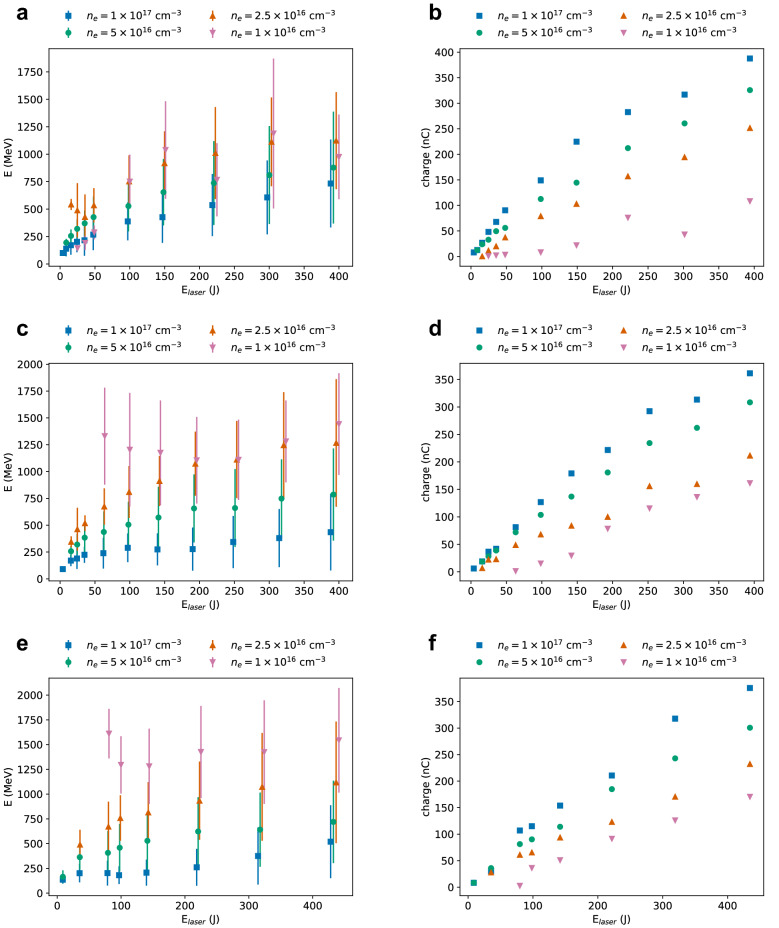
(**a, c, e**) Mean energy and (**b, d, f**) charge of electron beams produced in a pre-ionised plasma of varying density by a laser pulse with duration of 500 fs (FWHM), wavelength of ${10.6}\,\upmu \hbox {m}$, while varying energy, and waist size of (**a, b**) ${100}\,\upmu \hbox {m}$, (**c, d**) ${200}\,\upmu \hbox {m}$ and (**e, f**) ${300}\,\upmu \hbox {m}$. Error bars correspond to the r.m.s. energy spread.

Figure 3 shows the mean energy (a, c, e) and charge (b, d, f) of electron beams generated in a pre-ionised plasma with density between ${1 \times 10^{16}}\,{\hbox {cm}^{-3}}$ and ${1 \times 10^{17}}\,{\hbox {cm}^{-3}}$ by a laser pulse with 500 fs duration, energy between 2 J and 440 J (4–850 TW), and beam waist of ${100}\,\upmu \hbox {m}$ (a, b), ${200}\,\upmu \hbox {m}$ (b, c) and ${300}\,\upmu \hbox {m}$ (d, e). As the laser energy increases, electron beams with energy up to about 1.5 GeV and charge up to about 350 nC are obtained, observing similar trends for most plasma densities and laser waist sizes considered. The energy conversion efficiency is typically 10–70%, also increasing with laser energy. Typically, the bunch length increases from about ${10}\,\upmu \hbox {m}$ to ${150}\,\upmu \hbox {m}$, the divergence from 10 mrad to 50 mrad and the normalised emittance from ${10}\,{\pi\, \hbox {mm}\,\hbox{mrad}}$ to ${1000}\,{\pi\, \hbox {mm}\,\hbox{mrad}}$. The acceleration length varies from about 10–20 mm for $n_e={1 \times 10^{17}}\,{\hbox {cm}^{-3}}$ to 100–200 mm for $n_e={1 \times 10^{16}}\,{\hbox {cm}^{-3}}$. In some cases, increasing the laser energy can result in late injection of a second bunch, which causes the mean electron energy to decrease. This is particularly evident for a plasma density of ${1 \times 10^{16}}\,{\hbox {cm}^{-3}}$. A more detailed discussion of the scaling laws is provided in the next section.

For a beam waist of ${100}\,\upmu \hbox {m}$, the best operating plasma densities are ${5 \times 10^{16}}\,{\hbox {cm}^{-3}}$ and higher. For plasma densities lower than ${2.5 \times 10^{16}}\,{\hbox {cm}^{-3}}$ and low laser energies, self-focusing cannot balance diffraction and acceleration stops after relatively short distances. Background plasma electrons are also not fully evacuated from the accelerating structure, causing the injected bunch to drive an additional blowout, which degrades the beam quality. The resulting electron beam has a relative low energy and charge. For example, a laser pulse with an energy of 50 J ($a_0=7$) produces a 280 MeV electron beam with 3 nC charge in a plasma with a density of ${1 \times 10^{16}}\,{\hbox {cm}^{-3}}$ and length of 47 mm. On the other hand, a laser pulse with the same parameters propagating in a plasma with parabolic radial profile produces an electron beam with an energy of 900 MeV and a charge of 25 nC in a length of 150 mm. However, for higher laser energies and larger waist sizes, electron beams with mean energy up to 1.5 GeV and 150 nC charge are obtained in a uniform plasma for this density and no significant differences are observed when using external guiding.

**Figure 4:**
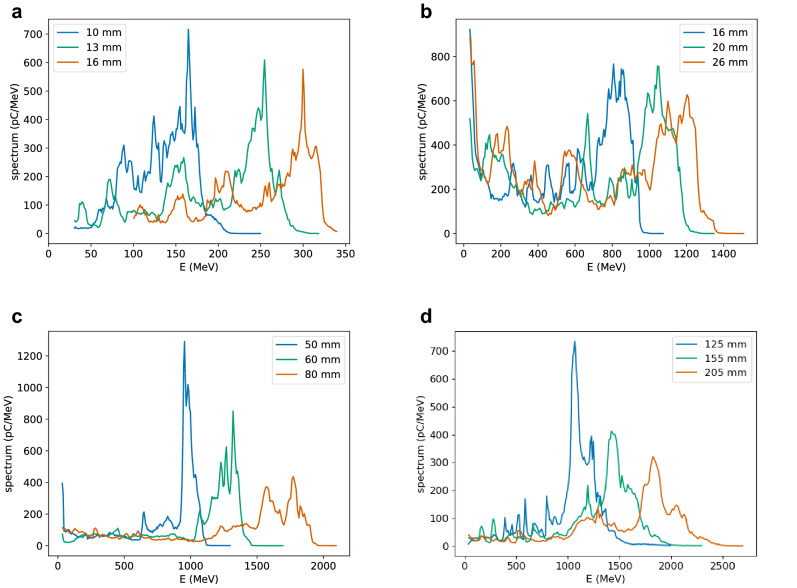
Selected electron energy spectra at different stages of acceleration produced in a pre-ionised plasma by a laser pulse with duration of 500 fs (FWHM) and wavelength of ${10.6}\,\upmu \hbox {m}$. The laser waist size, normalised vector potential and plasma density are: (**a**) $w_0={100}\,\upmu \hbox {m}$, $a_0=5$ ($E={25}\,{\hbox {J}}$), $n_e={1 \times 10^{17}}\,{\hbox {cm}^{-3}}$; (**b**) $w_0={100}\,\upmu \hbox {m}, a_0=20$ ($E={400}\,\hbox{J}$), $n_e={1 \times 10^{17}}\,{\hbox {cm}^{-3}}$; (**c**) $w_0={200}\,\upmu \hbox {m}, a_0=10$ ($E={400}\,\hbox{J}$), $n_e={2.5 \times 10^{16}}\,{\hbox {cm}^{-3}}$; (**d**) $w_0={300}\,\upmu \hbox {m}, a_0=7$ ($E={430}\,{\hbox {J}}$), $n_e={1 \times 10^{16}}\,{\hbox {cm}^{-3}}$.

Electron energy spectra for selected laser and plasma parameters are presented in Fig. 4. For a plasma density of ${1 \times 10^{17}}\,{\hbox {cm}^{-3}}$, a laser pulse with a waist size of ${100}\,\upmu \hbox {m}$ and energy of 100 J ($a_0=10$) produces a 210 MeV electron beam with 41% r.m.s. energy spread, 23 nC charge and ${60}\,\upmu \hbox {m}$ bunch length after 16 mm propagation (Fig. 4a). If only the high-energy peak is selected (${240}\,{\hbox {MeV}}<\hbox {E}<{340}\,\hbox {MeV}$), the mean energy is 290 MeV, with 7% r.m.s. energy spread and 18 nC charge. If the laser energy is increased to 400 J ($a_0=20$), the high-energy peak (${0.8}\,\hbox {GeV}<\hbox {E}<{1.4}\,\hbox {GeV}$) has a mean energy of 1.1 GeV, with 12% r.m.s. energy spread and 180 nC charge after 26 mm propagation (Fig. 4b). For $n_e={2.5 \times 10^{16}}\,{\hbox {cm}^{-3}}$, $w_0={200}\,\upmu \hbox {m}$ and $E_{laser}={400}\,{\hbox {J}}$ ($a_0=10$), the high-energy peak (${1.2}\,\hbox {GeV}<\hbox {E}<{2}\,\hbox {GeV}$) has a mean energy of 1.6 GeV with 11% r.m.s. energy spread and 130 nC charge after 80 mm propagation (Fig. 4c). The electron beam energy can be increased further using $n_e={1 \times 10^{16}}\,{\hbox {cm}^{-3}}$, $w_0={300}\,\upmu \hbox {m}$ and $E_{laser}={430}\,{\hbox {J}}$ ($a_0=7$), which results in a high-energy peak (${1}\,\hbox {GeV}<E<{2.5}\,\hbox {GeV}$) with a mean energy of 1.7 GeV, 19% r.m.s. energy spread and 145 nC charge after 205 mm propagation (Fig. 4d). In some cases the energy spread is narrower if acceleration is stopped before dephasing.

**Figure 5:**
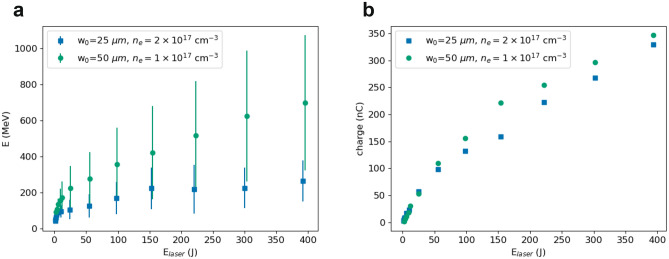
(**a**) Mean energy and (**b**) charge of electron beams produced in a pre-ionised plasma of density ${1 \times 10^{17}}\,{\hbox {cm}^{-3}}$ and ${2 \times 10^{17}}\,{\hbox {cm}^{-3}}$ by a laser pulse with duration of 500 fs (FWHM), wavelength of ${10.6}\,\upmu \hbox {m}$, varying energy, and waist size of ${25}\,\upmu \hbox {m}$ and ${50}\,\upmu \hbox {m}$. Error bars correspond to the r.m.s. energy spread.

At high plasma densities, the accelerator can be operated using waist sizes smaller than ${100}\,\upmu \hbox {m}$, as shown in Fig. 5. For a beam waist of ${25}\,\upmu \hbox {m}$ and a plasma density of ${2 \times 10^{17}}\,{\hbox {cm}^{-3}}$, electrons are produced with energy up to about 260 MeV and charge up to about 330 nC in a 6–13 mm long plasma. If the beam waist is increased to ${50}\,\upmu \hbox {m}$, the maximum energy is about 650 MeV with a charge of 350 nC in a 25 mm long plasma with density of ${1 \times 10^{17}}\,{\hbox {cm}^{-3}}$. At lower plasma densities, however, the accelerator performance is limited by diffraction. For example, for $n_e={2.5 \times 10^{16}}\,{\hbox {cm}^{-3}}$ a laser pulse with a waist size of ${50}\,\upmu \hbox {m}$ and an energy of 12 J ($a_0=7$) produces a 170 MeV electron beam with 60% r.m.s. energy spread and 0.5 nC charge in a length of 61 mm. When using external guiding, the energy increases to 290 MeV with 11 nC charge in a length of 72 mm.

### Scaling laws

We have compared the results of our PIC simulations with the predictions of analytical formulas based on phenomenological^30^ and similarity^31^ theories. These simplified models use laser and plasma parameters that aim to enhance the accelerator performance and minimise the evolution of the laser pulse and accelerator cavity, which is assumed to be approximately spherical. Reference^30^ proposes the matched condition

1 $$\begin{aligned} k_p R \approx k_p w_0 \approx 2 \sqrt{a_0}, \end{aligned}$$

with *R* the bubble radius, whereas reference^31^ uses

2 $$\begin{aligned} k_p R \approx k_p w_0 \approx 1.12 \sqrt{a_0}. \end{aligned}$$

Here we perform simulations for a wide range of parameters that are not necessarily matched and find that $a_0$ and bubble size change significantly during propagation in the plasma. The bubble expands longitudinally, occasionally more than doubling in size, whereas its transverse size grows by more than 50%. Fits of the simulation results indicate that the average bubble size is approximately

3 $$\begin{aligned} R \approx R_0 \sqrt{\frac{\lambda _0[\,\upmu \hbox {m}]}{10.6}} \, P[\hbox {TW}]^{1/4} \left( \frac{10^{18}}{n_e[{\hbox {cm}^{-3}}]}\right) ^{1/3}, \end{aligned}$$

with $R_0\approx {17}\,\upmu \hbox {m}$ for the transverse bubble size and $R_0\approx {22}\,\upmu \hbox {m}$ for the longitudinal size. The dependence on $w_0$ has been neglected because it is weak for $w_0 \gtrapprox {50}\,\upmu \hbox {m}$. For low plasma densities this formula slightly overestimates the bubble size.

The electron beam energy is expected to scale approximately as^30^

4 $$\begin{aligned} E \approx \epsilon _{av} k_p L_{acc} m_e c^2, \end{aligned}$$

where $m_e$ is the electron mass, $\epsilon _{av}$ the average accelerating field and $L_{acc}$ the acceleration length. Reference ^30^ uses $\epsilon _{av} = \sqrt{a_0}/2$ and

5 $$\begin{aligned} L_{acc} \approx \frac{2}{3} \frac{\omega _0^2}{\omega _p^2} R, \end{aligned}$$

which is valid when acceleration is limited by dephasing. This assumption is compatible with most of our results, except for simulations limited by diffraction, such as for low plasma densities and low laser energies. Equation (4) can be rewritten using Eq. 3 (with $R_0={19.5}\,\upmu \hbox {m}$) and Eq. 5, obtaining

6 $$\begin{aligned} E[\hbox {MeV}] \approx 52 \frac{10.6}{\lambda _0[\,\upmu \hbox {m}]} \frac{\sqrt{P[\hbox {TW}]}}{\sqrt{w_0[\,\upmu \hbox {m}]}} \left( \frac{10^{18}}{n_e[{\hbox {cm}^{-3}}]}\right) ^{5/6}, \end{aligned}$$

which reproduces our simulation results well. The predicted energies are typically higher than the mean values shown in Figs. 3 and 5, but these are weighted down by the low-energy tail and do not represent the energy of the quasi-monoenergetic peaks. Further details on the dependence on the laser waist size and pulse duration are provided in the following sections.

Reference^30^ estimates that under matched conditions (Eq. 1) the number of accelerated electrons is

7 $$\begin{aligned} N \approx 2.5\times 10^{9} \frac{\lambda _0[\upmu \hbox {m}]}{0.8} \sqrt{\frac{P[\hbox {TW}]}{100}}. \end{aligned}$$

Reference^31^ predicts a similar scaling, but with a different proportionality factor and matched condition (Eq. 2). The derivation of Eq. 7 is based on the assumption that electrons absorb all the electromagnetic field energy contained in the accelerating cavity and an equal amount of plasma kinetic energy, which both scale as $(k_p R)^5$. Such a strong dependence on *R* implies that small changes in bubble size produce large variations in charge. In our simulations, the bubble is often longitudinally elongated and its size and shape evolve significantly during propagation in the plasma. Eq. 7 underestimates the charge and does not reproduce the scaling with the laser and plasma parameters that have been observed. Most results presented here agree with the formula

8 $$\begin{aligned} N \approx 9\times 10^{10} \frac{\lambda _0[\upmu \hbox {m}]}{10.6} E_{laser}^\delta [\hbox {J}] \sqrt{\frac{n_e[{\hbox {cm}^{-3}}]}{10^{18}}}, \end{aligned}$$

where the parameter $\delta$ depends on the plasma density and laser waist size. Fits of the simulation results indicate that $\delta \approx 3/4$ for $n_e \gtrapprox {2.5 \times 10^{16}}\,{\hbox {cm}^{-3}}$ and $w_0 \gtrapprox {100}\,\upmu \hbox {m}$. For $w_0 < {100}\,\upmu \hbox {m}, \delta$ decreases, down to about 1/2 for $w_0 ={25}\,\upmu \hbox {m}$.

The bunch length scales as

9 $$\begin{aligned} \sigma _z[\upmu \hbox {m}] \approx 33\, E_{laser}^{\delta _z}[\hbox {J}], \end{aligned}$$

where $\delta _z\approx \delta ^{1/3}$. The transverse beam size follows a similar scaling with energy, but it also increases with the waist size and plasma density. The beam performs betatron oscillations and is typically larger along the laser polarisation plane. Interaction with the laser can introduce additional modulations, especially for long pulse durations. The charge density is also difficult to estimate, due to bunch sub-structure.

In general, we found that the scaling laws presented here reproduce results obtained for densities $n_e \gtrapprox {2.5 \times 10^{16}}\,{\hbox {cm}^{-3}}$, $w_0\gtrapprox {50}\,\upmu \hbox {m}$ and laser pulse durations between 250 fs and 750 fs. They are not accurate close to the threshold for injection, and also when self-guiding is less effective, such as for $n_e={1 \times 10^{16}}\,{\hbox {cm}^{-3}}$ and $T_{FWHM}={125}\,\hbox{fs}$. A comprehensive study of the bunch structure, transverse beam size and emittance would require simulations with a larger number of azimuthal modes and higher resolution than performed here.

### Dependence on laser beam waist

Simulation results presented in the previous sections indicate that nC-level electron beams with energies up to 1–2 GeV can be produced for a wide range of laser waist sizes. Previous work^30,31^ targeting near-infrared lasers suggest that the accelerator performance is optimal when the laser waist and bubble size are matched (Eq 1, 2). Here we directly investigated the dependence of the electron beam properties on $w_0$ for a CO_2_ laser pulse with fixed power.

**Figure 6:**
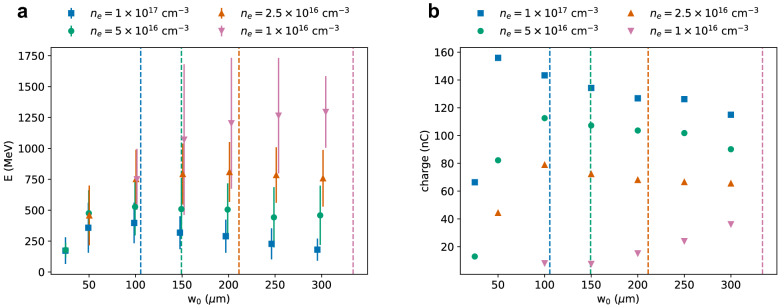
(**a**) Mean energy and (**b**) charge of electron beams produced in a pre-ionised plasma of varying density by a laser pulse with duration of 500 fs (FWHM), energy of 100 J, wavelength of ${10.6}\,\upmu \hbox {m}$ and varying waist size. Vertical lines mark the plasma wavelength $\lambda _p$. Error bars correspond to the r.m.s. energy spread.

Figure 6 shows the mean energy (a) and charge (b) of electron beams generated in a uniform plasma with a density between ${1 \times 10^{16}}\,{\hbox {cm}^{-3}}$ and ${1 \times 10^{17}}\,{\hbox {cm}^{-3}}$ by a laser pulse with 500 fs duration, 100 J energy ($P={200}\,\hbox {TW}$) as a function of beam waist size. The corresponding $a_0$ ranges from 3.3 to 40. For all densities, the highest electron energy is obtained when $w_0 \approx \lambda _p$, which is marked by the vertical lines in the plot. For the chosen laser power, the average bubble size is also close to $\lambda _p$ (Eq. 3). The charge follows a different trend, as shown in Fig. 6b. It drops sharply for small $w_0$, when the accelerator performance is limited by diffraction, and it decreases slowly for large $w_0$ and $n_e\gtrapprox {2.5 \times 10^{16}}\,{\hbox {cm}^{-3}}$. The efficiency has a maximum of about 55% close to $w_0={100}\,\upmu \hbox {m}$ for densities down to ${2.5 \times 10^{16}}\,{\hbox {cm}^{-3}}$, and is about 10% for ${1 \times 10^{16}}\,{\hbox {cm}^{-3}}$ and the selected $w_0$ range. For $n_e\gtrapprox {5 \times 10^{16}}\,{\hbox {cm}^{-3}}$ the spot size, divergence and emittance are slightly smaller when $w_0 \approx \lambda _p$, but for lower densities the dependence on $w_0$ is weaker and sometimes better results are obtained when $w_0 < \lambda _p$. Simulations performed for higher laser energy, when the bubble size $R>\lambda _p$, predict that the maximum electron energy is still obtained when $w_0 \approx \lambda _p$, but the drop in charge for small beam waists is reduced.

### Dependence on laser pulse duration

Most of the results presented so far have been obtained for a laser pulse duration $T_{FWHM}={500}\,\hbox{fs}$, but self-guiding in a plasma is expected to be more efficient when $T_{FWHM} \approx \lambda _p/c$, as discussed in the introduction. Here we study the dependence of the electron beam properties on the pulse duration for a fixed beam waist, adjusting the laser energy to keep the power constant.

**Figure 7:**
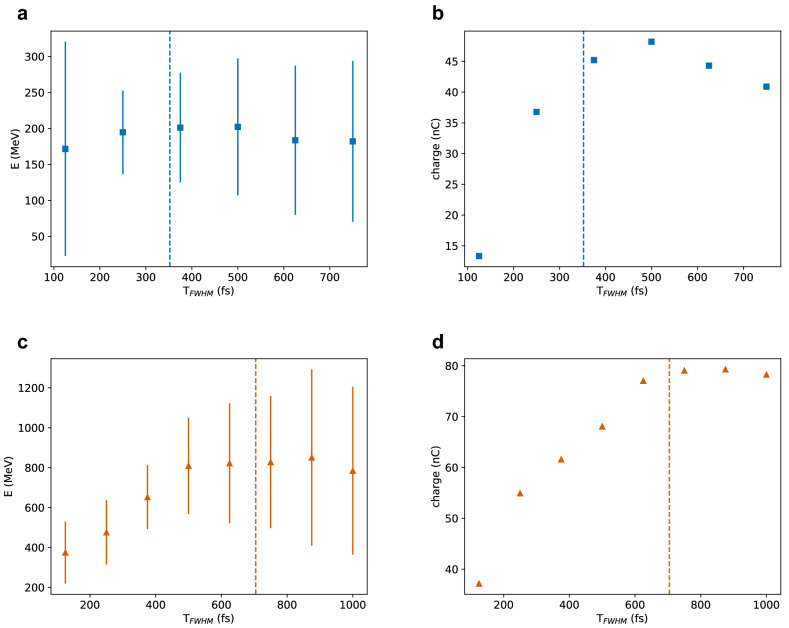
(**a,c**) Mean energy and (**b,d**) charge of electron beams produced in a pre-ionised plasma by a laser pulse with wavelength of ${10.6}\,\upmu \hbox {m}$, $a_0=5$ and varying duration. The plasma density and laser waist size are: (**a,b**) ${1 \times 10^{17}}\,{\hbox {cm}^{-3}}$ and ${100}\,\upmu \hbox {m}$; (**c,d**) ${2.5 \times 10^{16}}\,{\hbox {cm}^{-3}}$ and ${200}\,\upmu \hbox {m}$. Vertical lines mark the plasma wavelength $\lambda _p$. Error bars correspond to the r.m.s. energy spread.

Figure 7 shows the mean energy and charge of electron beams generated by a laser pulse with varying duration and $a_0=5$. In Fig. 7a and b the waist size is ${100}\,\upmu \hbox {m}$, which is matched to the plasma density of ${1 \times 10^{17}}\,{\hbox {cm}^{-3}}$, as shown in the previous section. The corresponding laser power is 50 TW, with energy between 6 J and 24 J. The laser beam remains focused past the dephasing length for all pulse durations considered, resulting in approximately the same final electron energy, as shown in Fig. 7a. The mean energy is slightly higher and the energy spread narrower when $T_{FWHM}\approx \lambda _p/c$, which is marked by the vertical line in the plot. The charge increases with pulse duration, because of the higher laser energy, but slowly decreases for $T_{FWHM} > \lambda _p/c$. The bunch length, transverse beam size, emittance and divergence also increase with pulse duration. The efficiency is peaked at 250 fs and ranges from 35 to 50%. For long pulse durations the front of the electron bunch can interact with the back of the laser pulse, which causes beam degradation, but can boost the emission of betatron radiation^32^.

Figure 7c and d shows the mean energy and charge generated in a plasma with density of ${2.5 \times 10^{16}}\,{\hbox {cm}^{-3}}$ by a laser pulse with matched spot size $w_0={200}\,\upmu \hbox {m}$ and $a_0=5$, which corresponds to a power $P={190}\,\hbox{TW}$ and energy between 25 J and 200 J. For short pulse durations, the accelerator performance is limited by diffraction and the electron energy increases linearly with $T_{FWHM}$, until dephasing is reached at about 500 fs. The charge forms a plateau at $T_{FWHM} \approx \lambda _p/c$. The efficiency is peaked at 500 fs and ranges from 44 to 54%.

## Discussion

We have performed PIC simulations of a LWFA driven by a ${10.6}\,\upmu \hbox {m}$ laser emitting sub-picosecond pulses with powers of 10s–100s TW. Results indicate that electron beams with 100s MeV to GeV energy and 1–100s nC charge can be produced in a pre-ionised uniform plasma with density of $10^{16}$–$10^{17}\,{\hbox {cm}^{-3}}$, using no external guiding, with a laser to electron beam efficiency that can exceed 50%. For example, a 20 TW CO_2_ laser emitting pulses with 500 fs duration and 10 J energy focused to a waist size of ${50}\,\upmu \hbox {m}$ into a plasma with density of ${1 \times 10^{17}}\,{\hbox {cm}^{-3}}$ and length of 16 mm produces an electron beam with mean energy of 230 MeV with 4% r.m.s. energy spread, 4 nC charge and 14 mrad r.m.s. divergence, selecting only the high-energy peak. If the laser energy is increased to 100 J ($P={200}\,\hbox {TW}$), the spot size to $w_0={100}\,\upmu \hbox {m}$, and the plasma length is set to 20 mm, the mean energy of the selection is 560 MeV with 7% r.m.s. energy spread, 50 nC charge and 15 mrad r.m.s. divergence. If the laser energy is further increased to 400 J ($P={800}\,\hbox {TW}$), with $w_0={200}\,\upmu \hbox {m}$, $n_e={2.5 \times 10^{16}}\,{\hbox {cm}^{-3}}$ and a plasma length of 86 mm, the mean energy of the selection is 1.6 GeV with 12% r.m.s. energy spread, 140 nC charge and 6 mrad r.m.s. divergence. Such charge levels are significantly higher than what is expected from near-infrared lasers of similar power, although the bunch length and emittance can be larger. Nevertheless, currents of 100s kA are achievable.

We have shown that a wide range of laser waist sizes and pulse durations can be employed, but the electron beam energy is maximised when $w_0 \approx \lambda _p$ and $T_{FWHM} \approx \lambda _p/c$. The charge, on the other hand, is typically maximised for slightly smaller waist sizes and longer pulse durations. However, self-guiding is often less effective for waist sizes smaller than ${50}\,\upmu \hbox {m}$ and pulse durations shorter than 250 fs, resulting in lower electron beam energies and charges, especially for low plasma densities and low laser energies. Under these conditions, external guiding can significantly enhance the electron beam energy. On the other hand, pulse durations approaching 1 ps can degrade the electron beam quality due to interaction with the laser field, but could potentially boost the emission of betatron radiation.

**Figure 8:**
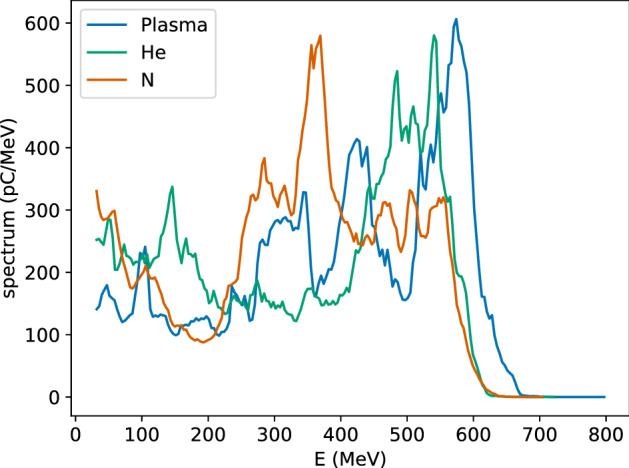
Energy spectra of an electron beam produced by a laser pulse with wavelength of $10.6\,\upmu \hbox {m}$, duration of 500 fs (FWHM), $a_0=10$ and $w_0={100}\,\upmu \hbox {m}$ in a pre-ionised plasma of density ${1 \times 10^{17}}\,{\hbox {cm}^{-3}}$, neutral helium and nitrogen. The gas density is chosen to produce a plasma with density of ${1 \times 10^{17}}\,{\hbox {cm}^{-3}}$, assuming full ionisation.

Results presented so far have been obtained for a pre-ionised plasma, but simulations performed using neutral gas indicate that a CO_2_ laser can fully ionise the atoms. For example, Fig. 8 shows electron spectra generated by a 500 fs laser pulse with a waist size of ${100}\,\upmu \hbox {m}$ and energy of 100 J ($a_0=10$) in a uniform pre-ionised plasma and neutral gas (helium and nitrogen). Assuming full ionisation of the atoms, the resulting plasma density is ${1 \times 10^{17}}\,{\hbox {cm}^{-3}}$ in all cases. An electron beam is produced with mean energy of 390 MeV in pre-ionised plasma, 345 MeV in helium and 330 MeV in nitrogen. The r.m.s. energy spread is between 40 and 50% and the charge about 150 nC in all cases. The acceleration length is similar in helium and in pre-ionised plasma, but about 5 mm longer in nitrogen. As an alternative to neutral gas targets, plasma discharge waveguides^29^ or heater pulses^33^ can also be considered, especially for low laser powers.

**Figure 9:**
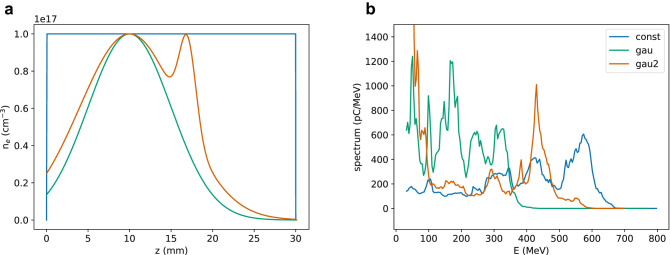
(**a**) Plasma density distribution and (**b**) energy spectra of electron beams produced in a pre-ionised plasma with peak density of ${1 \times 10^{17}}\,{\hbox {cm}^{-3}}$ by a laser pulse with wavelength of ${10.6}\,\upmu \hbox {m}$, duration of 500 fs (FWHM), energy of 100 J ($a_0=10$) and $w_0={100}\,\upmu \hbox {m}$.

Here, no attempts have been made to optimise parameters such as emittance, energy spread and bunch duration, but techniques such as down-ramp injection^34^ and density modulation^35^ could be used to control injection and the properties of the beam. Moreover, techniques based on ionisation injection^36^ could further boost the beam charge and efficiency. As an example, Fig. 9 shows the energy spectra generated by a 500 fs pulse with a waist size of ${100}\,\upmu \hbox {m}$ and energy of 100 J ($a_0=10$) in a pre-ionised plasma for three different profiles (Fig 9a). For a uniform distribution, the dephasing length is about 20 mm, resulting in the production of an electron beam with mean energy of 390 MeV with 40% r.m.s. energy spread. The second profile considered is Gaussian, with centre at $z={10}\,{\hbox {mm}}$ and $\sigma _z={6}\,{\hbox {mm}}$. The acceleration length is shorter and the mean energy is 190 MeV with 50% r.m.s. energy spread. Adding a Gaussian bump centred at $z={17}\,{\hbox {mm}}$, however, enables to increase the mean energy and reduce the chirp^37^, leading to the formation of a peak at 430 MeV with r.m.s. energy spread of 9% and charge of 55 nC in the energy selection ${350}\,\hbox {MeV}<E<{550}\,\hbox {MeV}$. Profiles of this type can be realised experimentally placing obstacles to partly obstruct the flow of a gas jet or using multiple stage gas cells.

With advances in CO_2_ laser technology, such as the development of compact and efficient optical pumped lasers^23^, LWFAs driven by CO_2_ lasers could be ideal for applications that require high-charge electron beams, such as industrial imaging, activation analysis, radioisotope production and radiotherapy. A bright tunable X-ray source could also be realised using Compton back-scattering^38^. An optically pumped CO_2_ laser driven LWFA would be efficient, compact, robust and could be made mobile. It would be complementary to widely used LWFAs driven by near-infrared Ti:sapphire lasers, which can produce beam with higher energy, lower emittance and energy spread, but also lower charge. An example of an application of a high-charge LWFA driven by CO_2_ lasers is as an accelerator for cancer treatment using FLASH radiotherapy^39^, which relies on irradiating tumours with doses higher than 10 Gy and exposure times of milliseconds or shorter. So far, most studies in this field have been conducted using electron beams with energy close to 10 MeV from clinical accelerators, which are only suitable for the treatment of superficial tumours. A LWFA driven by a 20 TW CO_2_ laser could generated 200 MeV electron beams with a charge of 20 nC and sub-picosecond bunch duration. Such a highly-penetrating beam would deposit a dose of 25 Gy in water, enabling the treatment of deep-seated tumours in the FLASH regime.

## Methods

### FBPIC simulations $(\lambda _0={10.6}\,\upmu \hbox {m})$

Particle-in-cell simulations have been performed using the quasi-3D code FBPIC^22^ to model a LWFA driven by a ${10.6}\,\upmu \hbox {m}$ laser. The laser beam is linearly polarised and has a temporal $\cos ^2$ shape with duration between 125 fs and 1 ps (FWHM of the intensity). The transverse profile is Gaussian, focused to a waist size $w_0$ between ${25}\,\upmu \hbox {m}$ and ${300}\,\upmu \hbox {m}$ (in vacuum) at the entrance of a pre-ionised uniform plasma preceded by a ${60}\,\upmu \hbox {m}$ long up-ramp. Simulations have also been performed using a plasma with radial density profile $n_r = n_e + a\,r^2$, where *a* has been varied between ${8 \times 10^{28}}\,{\hbox {m}^{-5}}$ and ${1.5 \times 10^{29}}\,{\hbox {m}^{-5}}$. The number of azimuthal ($\theta$) modes is 2 and the number of macro-particles per cell in longitudinal, radial and azimuthal direction is $n_z=2$, $n_r=2$ and $n_\theta =8$, with cubic particle shape. The box size has been changed depending on the laser and plasma parameters. Typically, the length in the longitudinal direction *z* is 1–1.5 mm and the radius is 0.6–1 mm. The resolution is about 0.4–${0.6}\,\upmu \hbox {m}$ in the longitudinal direction and 0.6–${1}\,\upmu \hbox {m}$ in the radial direction. A few simulations have also been performed using a higher longitudinal resolution of ${0.1}\,\upmu \hbox {m}$, observing no significant differences in the average values reported here. A few simulations performed using 3 azimuthal modes also produced similar results, except for small differences in the bunch structure. Most simulations use a radial boundary with perfectly-matched-layers and 256–512 damping cells.

### FBPIC simulations $(\lambda _0={0.8}\,\upmu \hbox {m})$

Particle-in-cell simulations have been performed using the code FBPIC to model a LWFA driven by a ${0.8}\,\upmu \hbox {m}$ laser. The set-up is similar to the previous section, but the number of macro-particles per cell is $n_z=2$, $n_r=2$ and $n_\theta =4$. A simulation has been performed for a laser waist size of ${50}\,\upmu \hbox {m}$ and a pulse duration of 250 fs using a box size of ${400}\,\upmu \hbox {m}$ in the longitudinal direction and ${300}\,\upmu \hbox {m}$ in the radial direction, with a resolution of ${0.04}\,\upmu \hbox {m}$ and ${1}\,\upmu \hbox {m}$, respectively. Simulations have also been performed for smaller waist sizes and shorter pulse durations, using a typical box size of ${180}\,\upmu \hbox {m}\times {160}\,\upmu \hbox {m}$ and resolution of ${0.036}\,\upmu \hbox {m}\times {0.3}\,\upmu \hbox {m}$. Simulations performed for wavelengths between $0.8\,\upmu \hbox {m}$ and $10.6\,\upmu \hbox {m}$ use a box size and number of macro-particles that has been gradually increased, until reaching the configuration described in the previous section.

## Data Availability

Data associated with research published in this paper is available at 10.15129/6bb91792-4de1-405f-9cee-57228fc49713.
